# Use of and Comorbidities Associated With Diagnostic Codes for COVID-19 in US Health Insurance Claims

**DOI:** 10.1001/jamanetworkopen.2021.24643

**Published:** 2021-09-08

**Authors:** Kai-Cheng Yang, Byungkyu Lee, Yong-Yeol Ahn, Brea L. Perry

**Affiliations:** 1 Luddy School of Informatics, Computing, and Engineering, Indiana University-Bloomington, Bloomington; 2 Department of Sociology, Indiana University-Bloomington, Bloomington

## Abstract

This quality improvement study assesses the comorbidities associated with COVID-19 diagnostic codes in US health insurance claims.

## Introduction

Accurate identification of COVID-19 diagnosis in patient medical records is essential for studies using administrative data to examine morbidity, mortality, and risk factors associated with COVID-19.^1^ Before April 1, 2020, the Centers for Disease Control and Prevention suggested using the existing *International Statistical Classification of Diseases, Tenth Revision, Clinical Modification* (*ICD-10-CM*) code B97.29 (other coronavirus as the cause of diseases classified elsewhere) as the primary diagnostic code for patients infected with COVID-19.^2^ On April 1, 2020, a new code U07.1 (2019-nCoV acute respiratory disease) was added to *ICD-10-CM*^3^ and was rapidly adopted by hospitals.^4^ Our study examined how nonhospital and hospital health care professionals have used these diagnostic codes in practice using a national medical claims data set in the US. We analyzed the comorbidities associated with COVID-19 diagnosis to assess the specificity of the legacy code and the importance of using both codes.

## Methods

In this quality improvement study, we used the deidentified Clinformatics Data Mart Database (Optum), which comprises commercial and Medicare Advantage health plans members who are similar to the US commercially insured population with respect to demographic characteristics.^5^ Our analytic sample contained the longitudinal medical records of 28 853 694 patients across the US from January 1, 2018, to September 30, 2020. We examined the frequency of B97.29 and U07.1 in 2020 to understand their adoption by hospital and nonhospital health care professionals. We only considered the first encounter to avoid double counts. Because the legacy code may be used for other coronavirus incidents, we identified the most common co-occurring diagnoses before and after January 1, 2020, and calculated the correlation of their frequency using the SciPy package in Python, version 1.5.2. The same analysis was conducted for U07.1 for comparison. This study was approved by the Indiana University institutional review board and followed the Standards for Quality Improvement Reporting Excellence (SQUIRE) reporting guideline. Owing to the use of deidentified patient data, the need for informed consent was waived by the institutional review board.^6^

## Results

Of the 18 975 615 patients (mean [SD] age, 47.9 [24.1] years; 9 832 556 women [51.8%]; 9 143 059 men [48.2%]) in the data set between January 1 and September 30, 2020, 26 414 (0.14%) were diagnosed with B97.29, and 279 066 (1.47%) were diagnosed with U07.1. The number of patients with a B97.29 code increased in March 2020 but rapidly diminished after the introduction of U07.1 (Figure). Although hospitals stopped using the legacy code shortly after the introduction of U07.1, some nonhospital health care professionals continued to use it (Figure). In 2020, 6 out of the 10 most frequent diagnoses that co-occurred with B97.29 were associated with COVID-19 according to the Centers for Disease Control and Prevention guideline,^3^ whereas in 2018 and 2019, only 1 out of 10 most frequent diagnoses that co-occurred with B97.29 was associated with COVID-19 (Table). The frequency of diagnostic codes that co-occurred with B97.29 in 2020 was more closely correlated with the frequency of those with U07.1 (Pearson *r*, 0.92; *P* < .001) than those with B97.29 in 2018 and 2019 (Pearson *r*, 0.58; *P* < .001). Using only U07.1 to identify patients with COVID-19 after April 1, 2020, missed 9714 patients diagnosed only with B97.29, consisting of 3.4% among 286 161 patients with either the legacy or new codes. However, the number of false positives due to screening B97.29 for patients with COVID-19 would be small, because the code’s drastic increase in 2020 can be attributed to COVID-19-related symptoms (Table).

**Figure.**
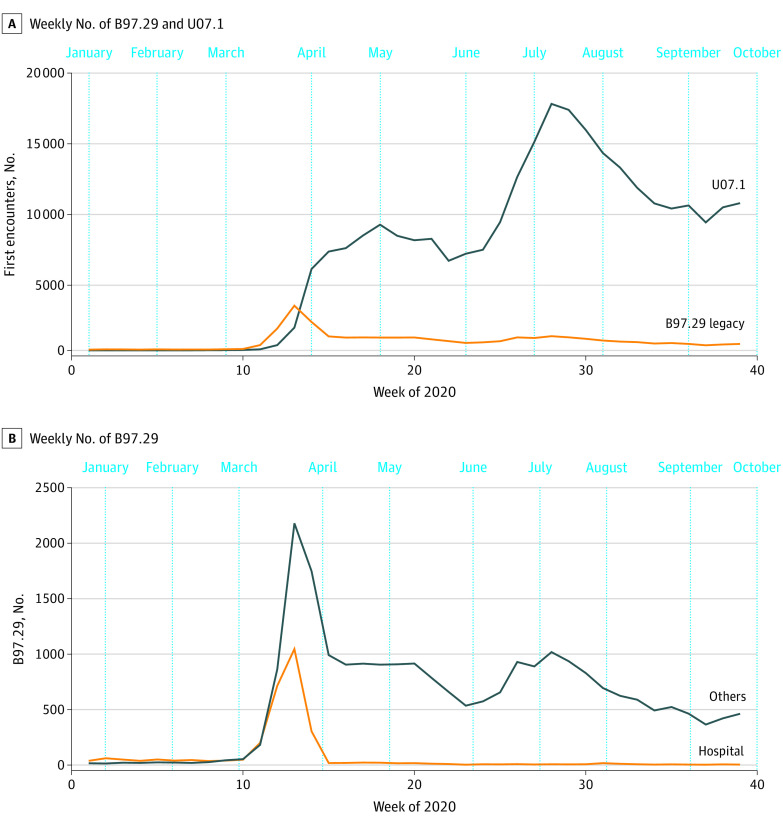
Trends of COVID-19 Diagnostic Codes Use From January 1 to September 30, 2020 (A) Weekly numbers of patients diagnosed with B97.29 and U07.1 codes. (B) Weekly numbers of B97.29 diagnoses from hospital and nonhospital health care professionals (individual, group practice, other facility, and unknown).

**Table.** Ten Most Common Diagnoses That Co-occurred With B97.29 and U07.1 in Different Time Periods

Order	B97.29 in 2018-2019 (n = 4290)			B97.29 in 2020 (n = 63 070)			U07.1 in 2020 (n = 1 285 888)		
	*ICD-10-CM*	Diagnosis name	No. (%)	*ICD-10-CM*	Diagnosis name	No. (%)	*ICD-10-CM*	Diagnosis name	No. (%)
1	I10	Essential (primary) hypertension	1035 (24.1)	J12.89^a^	Other viral pneumonia	14746 (23.4)	J12.89^a^	Other viral pneumonia	237485 (18.5)
2	E78.5	Hyperlipidemia, unspecified	998 (23.3)	U07.1^a^	2019-nCoV acute respiratory disease	8982 (14.2)	J96.01^a^	Acute respiratory failure with hypoxia	219697 (17.1)
3	J06.9	Acute upper respiratory infection, unspecified	848 (19.8)	J96.01^a^	Acute respiratory failure with hypoxia	8222 (13.0)	I10	Essential (primary) hypertension	177265 (13.8)
4	J44.1	Chronic obstructive pulmonary disease with (acute) exacerbation	761 (17.7)	R05^a^	Cough	6935 (11.0)	N17.9	Acute kidney failure, unspecified	95611 (7.4)
5	Z87.891	Personal history of nicotine dependence	730 (17.0)	Z20.828^a^	Contact with and (suspected) exposure to other viral communicable diseases	6533 (10.4)	J18.9	Pneumonia, unspecified organism	81150 (6.3)
6	K21.9	Gastro-esophageal reflux disease without esophagitis	717 (16.7)	I10	Essential (primary) hypertension	6478 (10.3)	E11.9	Type 2 diabetes mellitus without complications	73005 (5.7)
7	J96.01^a^	Acute respiratory failure with hypoxia	710 (16.6)	R50.9^a^	Fever, unspecified	6178 (9.8)	E78.5	Hyperlipidemia, unspecified	67506 (5.2)
8	Z79.899	Other long term (current) drug therapy	625 (14.6)	J18.9	Pneumonia, unspecified organism	4190 (6.6)	Z20.828^a^	Contact with and (suspected) exposure to other viral communicable diseases	58513 (4.6)
9	I25.10	Atherosclerotic heart disease of native coronary artery without angina pectoris	613 (14.3)	N17.9	Acute kidney failure, unspecified	3679 (5.8)	R05^a^	Cough	55469 (4.3)
10	N17.9	Acute kidney failure, unspecified	543 (12.7)	E78.5	Hyperlipidemia, unspecified	3518 (5.6)	R50.9^a^	Fever, unspecified	47375 (3.7)

Abbreviation: *ICD-10-CM*, *International Statistical Classification of Diseases, Tenth Revision, Clinical Modification*.

^a^ The diagnostic codes associated with COVID-19 according to the Centers for Disease Control and Prevention guideline.^3^

## Discussion

Using a hospital discharge data set, Kadri et al^4^ showed that the legacy code B97.29 was quickly replaced by U07.1, and its use decreased to prepandemic levels. Our quality improvement study confirmed their findings about hospitals by using large-scale medical claims data, but our results suggest that some nonhospital health care professionals have continued to use B97.29 for COVID-19 diagnoses in 2020. It is possible that patients diagnosed with B97.29 were infected with coronavirus diseases other than COVID-19, and our findings may not generalize to other data sources. However, future research on COVID-19 using claims data should consider both B97.29 and U07.1 when identifying patients with COVID-19 to avoid introducing a systematic bias across hospital and nonhospital health care professionals given the strong socioeconomic disparity in rates of COVID-19 testing and infection.
